# Cell‐Type‐Resolved Proteomics Reveals Distinct Immune and Metabolic Programs Between Primary and Recurrent Ascites in HGSOC

**DOI:** 10.1002/prca.70053

**Published:** 2026-08-18

**Authors:** Bo Ren, Kenneth Weke, Darryl Hardie, Sarah MacPherson, Javier A Alfaro, Julian J Lum, David R Goodlett

**Affiliations:** ^1^ School of Molecular Life Sciences University of Victoria Victoria British Columbia Canada; ^2^ Genome BC Proteomics Centre University of Victoria Victoria British Columbia Canada; ^3^ Deeley Research Centre BC Cancer Research Institute Victoria British Columbia Canada; ^4^ Basic and Translational Research BC Cancer Research Institute Victoria British Columbia Canada; ^5^ Department of Biochemistry & Microbiology University of Calgary Calgary Alberta Canada; ^6^ Riddell Centre for Cancer Immunotherapy Arnie Charbonneau Cancer Institute Cumming School of Medicine University of Calgary Calgary Alberta Canada; ^7^ Department of Computer Science University of Calgary Calgary Alberta Canada

**Keywords:** ascites fluid, HGSOC, immune cells, proteomics, tumor‐enriched cells

## Abstract

High‐grade serous ovarian carcinoma (HGSOC) frequently recurs after platinum–taxane therapy, yet recurrence‐associated changes within ascites remain unclear. We performed cell‐type‐resolved quantitative proteomics on paired ascites samples collected at primary diagnosis and at disease recurrence from three HGSOC patients, profiling unsorted cells alongside matched CD45^−^ tumor‐enriched and CD45^+^ immune‐enriched fractions. Conventional proteomic label‐free quantitative (LFQ) analysis yielded limited significant proteins due to inter‐patient heterogeneity. To address this, we applied Gene Set Enrichment Analysis (GSEA) to identify coordinated pathway‐level alterations associated with recurrence. Across all cellular compartments, recurrent samples exhibited consistent downregulation of interferon‐α/γ–associated pathways alongside enrichment of oxidative phosphorylation and stress‐adaptive metabolic programs. In the CD45^+^ immune compartment, these changes were accompanied by enhanced IL‐2/STAT5 signaling and reduced antigen‐presentation pathways. Together, these findings suggest that recurrent ascites is characterized by a shift toward oxidative metabolism and a more immunosuppressive microenvironment.

## 1 Introduction

High‐grade serous ovarian carcinoma (HGSOC) is the most lethal gynecologic malignancy, characterized by significant molecular and cellular heterogeneity that complicates treatment and drives frequent relapse [1]. Due to vague symptoms and the absence of effective screening, over 75% of HGSOC cases are diagnosed at an advanced stage, typically presenting with widespread peritoneal metastases and malignant ascites [2]. While first‐line platinum–taxane therapy initially yields high response rates, the majority of patients eventually develop chemotherapy‐resistant disease, including a subset classified as platinum‐resistant [3, 4, 5]. Recurrent HGSOC is notably more aggressive than the primary tumor as chemotherapy selects for drug‐tolerant persister and cancer stem cell populations, resulting in a poor median survival of only 12–24 months [6]. Clinically, the presence of ascites signals advanced disease and poor prognosis [7]. Malignant ascites is closely linked to the biology and clinical progression of HGSOC. Because HGSOC commonly disseminates throughout the peritoneal cavity, exfoliated tumor cells, immune cells, stromal cells, cytokines, metabolites, and extracellular vesicles accumulate within ascitic fluid, creating a dynamic tumor microenvironment rather than a passive fluid collection [8]. This biologically active microenvironment provides critical factors that drive tumor cell survival and proliferation [9]. As a selective niche, ascites fosters metastatic phenotypes and facilitates therapeutic escape in ovarian cancer through several interconnected mechanisms [10]. Ascitic fluid supplies soluble growth factors, cytokines, extracellular vesicles, and matrix‐remodeling components that promote spheroid formation, epithelial–mesenchymal plasticity, and peritoneal implantation, while ascites‐associated immune and stromal populations establish an immunosuppressive, therapy‐protective environment that allows tumor cells to resist anoikis, evade immune clearance, and tolerate chemotherapy‐induced stress.

Ascites constitutes a complex ecosystem of heterogeneous non‐tumor cells, particularly immune populations [11]. Various subsets—including dysfunctional T cells, regulatory T cells, and myeloid‐derived suppressor cells—collectively blunt antitumor immunity and drive poor outcomes [12, 13, 14]. Specifically, M2‐like macrophages (CD163^+^) represent a major component associated with early recurrence and a poor prognosis [15]. This tolerogenic niche is further reinforced by soluble immunoregulatory mediators, including TGF‐β, IL‐10, and VEGF, as well as immunosuppressive metabolites such as CD73‐generated adenosine, lactate, and kynurenine. These factors can impair effector T‐cell proliferation, cytokine production, cytotoxic function, and survival, thereby contributing to immune dysfunction within ovarian cancer ascites [16, 17]. Consequently, this environment explains the historically limited efficacy of immunotherapy in ovarian cancer [5].

Mass spectrometry (MS)‐based proteomics captures the expressed genome and post‐translational modifications, providing a functional readout that complements genomics [18]. In ovarian cancer, proteomic studies have identified markers for therapeutic response [19], chemosensitivity mediators such as CT45 [20], and molecular subgroups not evident through genomic analysis [21]. Prior investigations characterized ascites fractions to explore metastasis and chemoresistance [22, 23, 24, 25], and some other studies using metabolomic and integrative profiling further revealed metabolic signatures and intercellular crosstalk associated with disease progression [26, 27]. However, because these studies primarily used unsorted samples, cell‐type‐specific signals are often obscured [28, 29]. Dissecting proteomic differences between tumor and immune cells is therefore essential to identify fraction‐resolved drivers of HGSOC recurrence.

Here, we present a quantitative MS‐based proteomic analysis of ascites‐derived cells from ovarian cancer patients, encompassing three complementary cell types: CD45^+^ immune cells, CD45^−^ non‐immune cells enriched for tumor cells, and the ascites from which they were sorted. This study leverages ascites fluid as a readily accessible tumor sample [8] and addresses the heterogeneity of HGSOC by profiling distinct cellular components. By analyzing ascites‐derived cells from paired primary and recurrent samples, we aimed to identify proteomic alterations at the fraction level associated with disease recurrence.

## 2 Methods

### 2.1 Patient Sample Acquisition and Processing

Patient ascites samples (IROC60, IROC65, and IROC126) and clinical data were obtained through the BC Cancer Tumor Tissue Repository (certified by the Canadian Tissue Repository Network) under institutional ethics approval (UBC/BC Cancer REB #H07‐00463) with informed consent or an ethics‐board waiver. For patients IROC60, IROC65, and IROC126, ascites was collected at two distinct clinical stages: before chemotherapy (designated as primary ascites) and after platinum/taxane‐based chemotherapy upon disease recurrence (designated as recurrent samples). Each draw was processed in parallel to generate three cellular fractions (unsorted, CD45^+^ immune, and CD45^−^ cells). The three patients (IROC60, IROC65, IROC126) constituted three independent biological cases. For each patient, a single ascites draw was divided into three equal‐volume aliquots and processed as technical triplicates (triplicates derived from the same draw, not separate paracenteses). Samples were de‐identified and stored in a certified biobank (BRC‐00290) until analysis. Ascites fluid was centrifuged at 500 × g for 10 min at 4°C to pellet the cellular fraction. Cells were resuspended in freezing medium consisting of 50% heat‐inactivated human AB serum (Sigma–Aldrich), 40% RPMI‐1640 (Thermo Fisher Scientific), and 10% dimethyl sulfoxide (DMSO), and gradually cryopreserved. Cryovials were stored in liquid nitrogen vapor phase until further processing. Detailed patient characteristics are provided in Supplementary Table S1.

### 2.2 Cell‐Type Enrichment and Isolation

Unless otherwise specified, all reagents and solvents were LC–MS grade (Fisher Chemical or Thermo Fisher Scientific). Cryopreserved ascites cell suspensions were rapidly thawed in a 37°C water bath and immediately diluted dropwise into 10 mL of ice‐cold culture medium to minimize osmotic shock. Cells were pelleted by centrifugation (500 × g, 5 min, 4°C), resuspended in cold medium, and passed through a 40 µm cell strainer to remove aggregates. All subsequent handling was performed on ice to preserve cellular integrity. Cells were counted (Trypan Blue exclusion), and the suspension was adjusted to 1 × 10^5^ cells/mL in magnetic‐activated cell sorting (MACS) buffer (PBS with 0.5% human AB serum and 2 mM EDTA) [17, 30]. An aliquot of ∼10,000 cells from each sample was set aside as an unsorted fraction, while the remaining cells were enriched for immune and tumor populations. The unsorted fraction represents the native cellular composition of ascites, which includes tumor cells, immune cells, and stromal‐associated populations present in the fluid microenvironment. CD45^+^ leukocytes were isolated by incubating the cell suspension with Human CD45 MicroBeads (Miltenyi Biotec) for 15 min at 4°C, then passing the sample through an LS magnetic column on a QuadroMACS separator (Miltenyi) according to the manufacturer's protocol. The flow‐through contained the CD45^−^ cells, enriching for tumor cells. This fractionation procedure was performed as previously described for ascites samples. The purity of the CD45^+^ and CD45^−^ fractions was not quantified by flow cytometry; accordingly, an equal number of cells (≈10 000) was collected independently from each fraction so that the proteomic input was normalized by cell number rather than by assumed purity.

After separation, cells in each fraction were pelleted (500 × g, 5 min) and resuspended in sterile 0.9% saline at 1 × 10^5^ cells/ml. Aliquots of 100 µL (≈10 000 cells) from the CD45^+^, CD45^−^, and unsorted fractions were transferred to low‐bind microcentrifuge tubes on ice. Each tube was brought to a total volume of 1 mL with cold saline, gently mixed, and then centrifuged again (500 × g, 5 min). Supernatants were removed entirely, and the cell pellets were immediately flash‐frozen in liquid nitrogen (at least 5 min) and stored at –80°C until protein extraction. Among these samples, patients IROC126, IROC65, and IROC60 who had undergone chemotherapy were processed into three cell fractions: unsorted cells, CD45^−^ cells, and CD45^+^ (immune) cells.

### 2.3 Proteomics Sample Preparation

**FIGURE 1:**
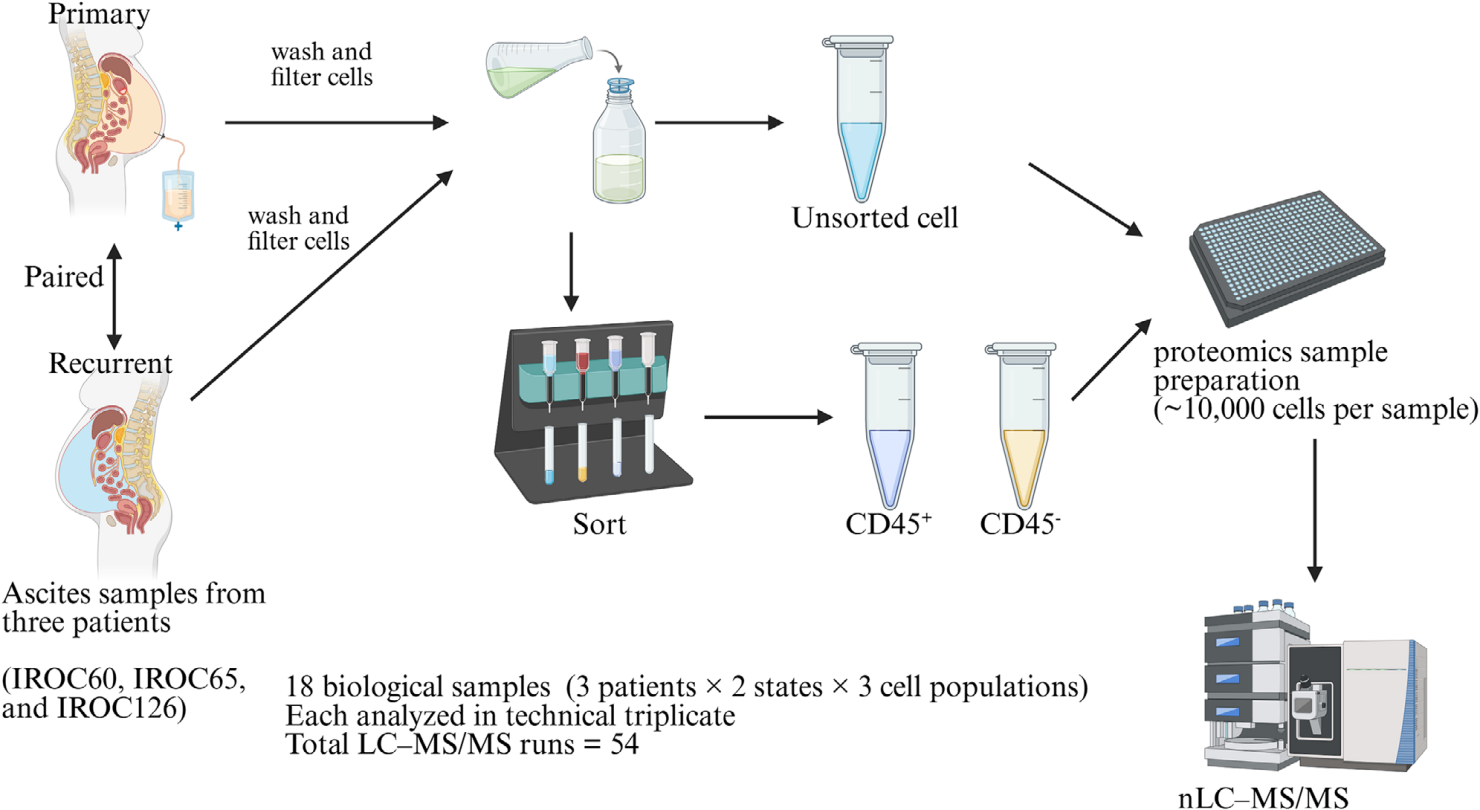
Workflow for paired cell‐type–resolved proteomic profiling of primary and recurrent ascites. Ascites samples were obtained from three patients at both primary diagnosis and disease recurrence, enabling within‐patient paired comparisons. After washing and filtration, cells were separated into unsorted, CD45^+^, and CD45^−^ populations. Low‐input proteomic analysis was performed on ∼10,000 cells per fraction using nanoLC–MS/MS. In total, 18 biological samples were generated, and each was analyzed in technical triplicate (54 LC–MS runs), allowing assessment of recurrence‐associated changes within specific cellular compartments.

Samples from three patients (primary and recurrent ascites) were fractionated into unsorted, CD45^+^, and CD45^−^ populations, yielding 18 biological samples, each analyzed in triplicate (total LC–MS runs = 54) (Figure 1). Proteomic sample preparation was adapted from a published single‐cell protocol [31] with modifications to accommodate ∼10 000 cells per sample. For lysis and digestion, cell pellets (∼10^4^ cells each) were thawed on ice and resuspended in PBS at ∼10 000 cells/µL. Lysis was carried out by probe sonication to disrupt cell membranes and release proteins. An aliquot of the resultant cell lysate containing ∼10 000 cell equivalents (1 µL) was then transferred into a well of a 384‐well plate. Proteins were digested using a modified version of the Promega Rapid Digestion Trypsin/Lys‐C kit (CAT# VA1061). The provided digestion buffer was replaced with 25 mM Tris–HCl (pH 8.0) containing 2 mM tris(2‐carboxyethyl)phosphine (TCEP). Lyophilized Trypsin/Lys‐C mix was reconstituted in water (to 0.1 µg/µL) and combined with the substitute buffer at a 1:15 (v/v) ratio. A 37.5 µL volume of this enzyme/buffer mixture was added to each well containing the 1 µL lysate (resulting in ∼38.5 µL total volume). Digestion was performed by incubating the plate in a water bath at 70°C for 3 h. After digestion, iodoacetamide (IAA) was added to each well to a final concentration of ∼70 mM (by adding 3.75 µL of 744 mM IAA solution) and incubated for 30 min at room temperature in the dark to alkylate cysteines. The reaction was quenched by the addition of 10% formic acid (FA). Peptide digests were desalted and concentrated using Oasis HLB solid‐phase extraction cartridges (10 mg sorbent, 30 µm; Waters), then lyophilized to dryness. Peptides were reconstituted in 40 µL of 2% acetonitrile / 0.1% FA and stored at 4°C until LC–MS analysis.

### 2.4 Nanoliquid Chromatography–Tandem Mass Spectrometry (nLC–MS/MS) Acquisition

Peptide samples (0.6 µg injection per sample) were analyzed by nano‐flow reverse‐phase LC–MS/MS on a Thermo Scientific EASY‐nLC 1000 system coupled to an Orbitrap Fusion Tribrid mass spectrometer (Thermo Fisher Scientific). Chromatographic separation was performed on an Acclaim PepMap 100 C18 pre‐column (100 µm ID × 2 cm, 5 µm, 100 Å) and an analytical nano‐column (75 µm ID × 150 mm, 3 µm, 100 Å; Thermo Fisher Scientific). Mobile Phase A was 2% acetonitrile / 0.1% FA in LC–MS grade water, and Mobile Phase B was 90% acetonitrile / 0.1% FA. After loading and equilibrating the columns (4 µL at 298 bar), peptides were eluted using a 140 min linear gradient from 5% to 40% B for 120 mins, followed by a column wash at 90% B and re‐equilibration for 20 mins. The flow rate was 300 nl/min.

The mass spectrometer was operated in positive ion data‐dependent acquisition mode. Nanospray ionization was achieved with a Nanospray Flex NG source (Thermo) using a spray voltage of 2.6 kV and capillary temperature of 275°C. The Orbitrap detector was used for survey scans (MS^1^), covering *m*/*z* 300–1500 at 120 000 resolving power (at *m*/*z* 200) with one microscan. Internal calibration was enabled using the polysiloxane ion at *m*/*z* 445.12002 as lock mass. The automatic gain control (AGC) target for MS^1^ was set to “Standard” (≈4 × 10^6^ ions, 100% setting) with an auto maximum injection time of 50 ms. Following each MS^1^scan, fragment ion (MS^2^) spectra were acquired for multiple precursors within a 3s cycle time. Precursor ions with charges 2–7 and intensities above 5 000 counts were sequentially isolated for MS^2^. High‐energy C‐trap dissociation (HCD) was used for fragmentation with a quadrupole isolation window of 1.6 Da. The Orbitrap Fusion was programmed to perform MS^2^ scans in the ion trap (rapid scan mode, normal mass range) with centroid detection. Monoisotopic precursor selection and isotope exclusion were enabled to prioritize peptide‐like features. Dynamic exclusion was applied to precursors after two occurrences within 30 s, for an exclusion duration of 15 s (±10 ppm mass tolerance). Ion trap MS^2^ scans were acquired with an AGC target of 10,000 ions (Standard setting) and a maximum injection time of 35 ms. A stepped normalized collision energy of 28%, 30%, 32% was used for HCD to maximize peptide coverage. Data were acquired using Thermo Fusion Tune 3.5 software in profile mode for MS^1^ and centroid mode for MS^2^.

### 2.5 Protein Identification and Data Analysis

Raw MS data were processed using the FragPipe pipeline (v21.1) with MSFragger [32] and Philosopher (v5.1.0). The tandem mass spectra were searched against a combined protein sequence database containing *Homo sapiens* (SwissProt taxonomy ID 9606, with 20453 entries, downloaded on August 3, 2025) and common contaminants. The default closed search MSFragger parameters were used (trypsin enzyme specificity, up to 2 missed cleavages, fixed cysteine carbamidomethylation, variable methionine oxidation and N‐terminal acetylation), with detailed settings provided in Supplementary File A. Peptide‐spectrum matches and protein identifications were filtered at 1% false discovery rate (FDR) at both peptide and protein levels [33]. Label‐free quantification was performed using MaxLFQ protein intensity calculation algorithm in IonQuant. Missing values in the protein intensity matrix were imputed using a k‐nearest neighbors (KNN) approach, which preserves underlying data structure and biological variance [34]. After imputation, LFQ intensity values were median‐normalized across samples to remove systematic technical biases, thereby facilitating accurate relative comparisons [35].

### 2.6 Differential Protein Expression Analysis (DEqMS)

Differential protein expression between experimental groups (e.g., recurrent vs. primary samples) was assessed using the DEqMS R package [36], which builds on the limma linear modeling framework for proteomics data. As described above, biological replicates corresponded to three independent patients, and technical replicates were averaged prior to statistical analysis to avoid pseudo‐replication. To account for inter‐patient variability and the paired structure of the data, analyses were performed using a patient‐blocked design, and intra‐patient correlation was estimated using the limma duplicate Correlation procedure. Empirical Bayes moderation was applied to obtain moderated t‐statistics and *p*‐values for each protein. Peptide count–adjusted *p*‐values generated by DEqMS were further corrected for multiple testing using the Benjamini–Hochberg method to control the false discovery rate (FDR) [37]. Proteins with *p*‐value < 0.05 and |log2 fold change| ≥ 1 were considered statistically significant for individual comparisons. Graphs were generated with GraphPad Prism (v10.4.0).

### 2.7 Gene Set Enrichment Analysis (GSEA)

Although GSEA was originally developed for transcriptomic data, rank‐based gene‐ and protein‐set enrichment approaches are now widely applied to quantitative mass spectrometry‐based proteomics to detect coordinated changes across functionally related proteins [38, 39]. Gene set enrichment analysis (GSEA) [40] was performed using the fgsea R package with gene sets from the Molecular Signatures Database (MSigDB) [41]. Specifically, the Hallmark gene sets (H database), computational gene sets (C4 database), immunologic signature gene sets (C7 database), and cell type signature gene sets (C8 database) were included in the analysis. Genes were ranked by signed DEqMS moderated t‐statistic to create a preranked list for each comparison, and GSEA was run with 10 000 permutations (nperm) while restricting to gene sets of 15–500 genes (minSize  =  15, maxSize  =  500). The normalized enrichment score (NES), nominal *p*‐value, and false discovery rate (FDR)‐adjusted *q*‐value were reported for each gene set. Gene sets with an FDR *q*‐value < 0.25 were considered significantly enriched, consistent with the threshold recommended in the original GSEA publication [40]. To confirm that the enrichment results were robust to the choice of ranking metric, we repeated GSEA using an alternative preranking based on sign(log_2_ fold change) × −log_10_(p) and obtained concordant NES across all fractions (Figure S1).

## 3 Results

For each patient, three technical replicates were generated from a single ascites sample collected at two time points: primary and recurrent. This analysis achieved robust and comparable proteome coverage in both primary and recurrent ascites. In the unsorted and CD45^−^ fractions, ∼1 500–2 500 proteins were identified per sample (Figures S2, S3), while the CD45^+^ (immune‐enriched) fraction yielded ∼1 200–2 000 proteins per sample (Figure S4). This coverage was stable across patients and disease states, forming the basis for downstream differential protein expression and biochemical pathway analyses.

**FIGURE 2:**
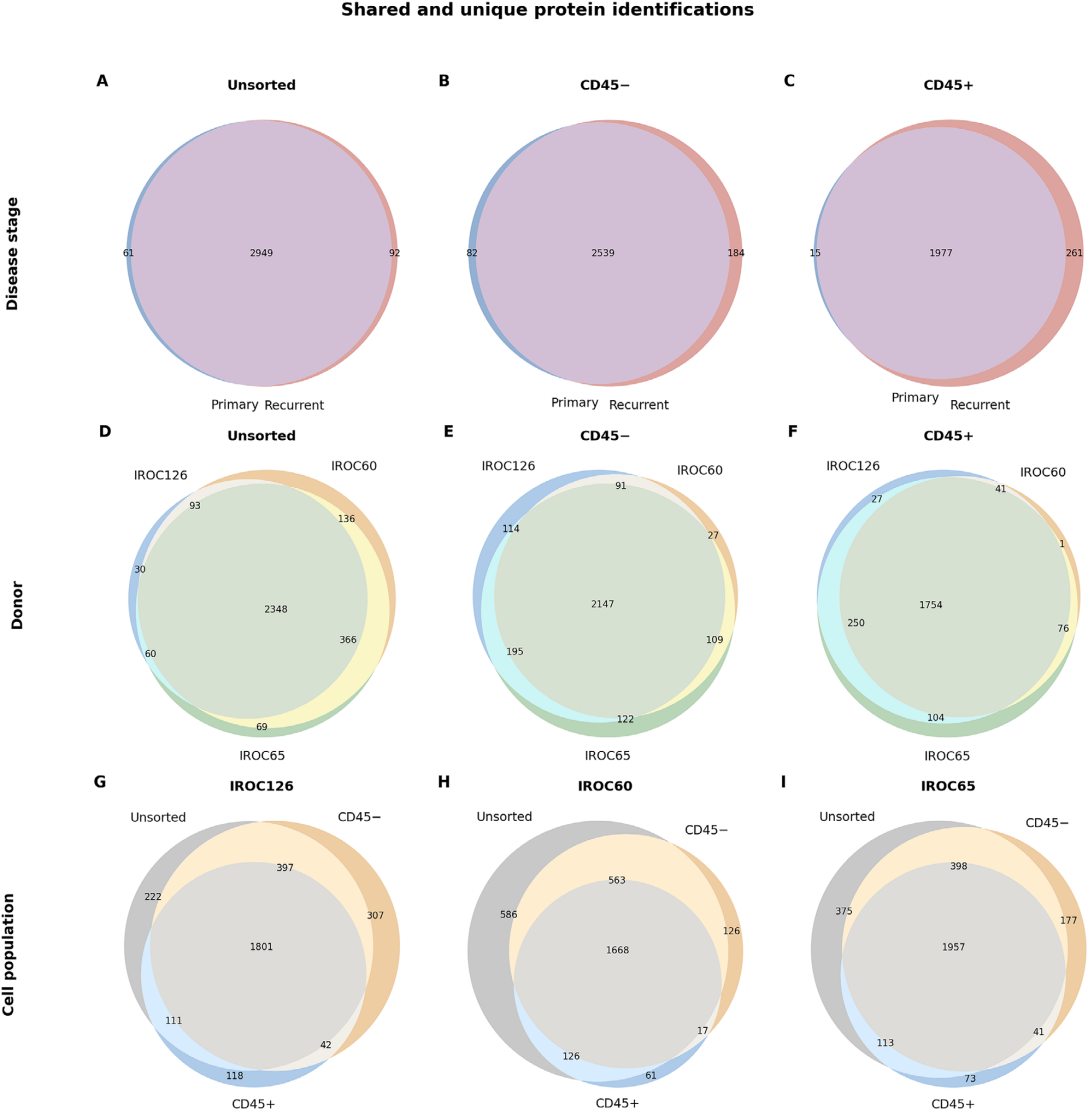
Shared and unique protein identifications across disease stage, donor, and cell population. Venn diagrams show the overlap of identified proteins (detected in at least one of three technical replicates, using unfiltered protein identifications) for three grouping variables. (A–C) Disease stage: proteins identified in primary versus recurrent ascites, shown separately for the unsorted (A), CD45^−^ tumor‐enriched (B), and CD45^+^ immune‐enriched (C) fractions. (D–F) Donor: proteins identified in each of the three patients (IROC126, IROC60, IROC65), shown separately for the unsorted (D), CD45^−^ (E), and CD45^+^ (F) fractions. (G–I) Cell population: proteins identified in the unsorted, CD45^−^, and CD45^+^ fractions, shown separately for patient IROC126 (G), IROC60 (H), and IROC65 (I). For each grouping, technical replicates were collapsed into a single set per sample, and the numbers indicate the protein counts in each region. The large, shared cores across all comparisons indicate a reproducible set of commonly identified proteins, with comparatively small stage‐, donor‐, and fraction‐specific subsets.

Consistent with this coverage, a comparison of protein identifications across disease stage, patient, and cellular fraction showed that most proteins were shared across all comparisons (Figure 2). In the unsorted fraction, for example, 2,949 proteins were common to primary and recurrent ascites (with 61 and 92 stage‐specific proteins, respectively), a core of 2,348 proteins was detected across all three patients, and 1,957 proteins were shared among the unsorted, CD45^−^, and CD45^+^ fractions in IROC65. Only small stage‐, patient‐, and fraction‐specific subsets were observed, indicating a reproducible core proteome underlying the group‐specific differences examined below.

**FIGURE 3:**
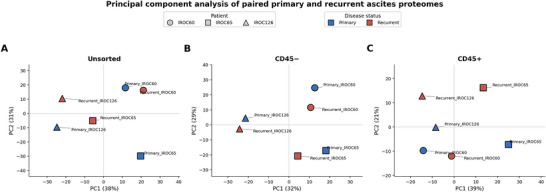
Principal component analysis of proteomic profiles from ascites‐derived cells showing separation of primary and recurrent samples. IROC60, IROC65, and IROC126 represent the three patient samples. Primary_IROC60, IROC65, IROC126 (blue) denote the Primary samples (collected before chemotherapy), and Recurrent_IROC60, IROC65, IROC126 (red) denote the corresponding Recurrent samples (collected upon disease recurrence).

Principal component analysis (PCA) of proteomic profiles from ascites‐derived cells showed that primary and recurrent samples from the same patient were separable, indicating recurrence‐associated differences (Figure 3). However, variation across patients showed strong inter‐patient heterogeneity. Consistent with this, a global primary‐versus‐recurrent comparison across unsorted cells, immune cells, and tumor‐enriched cells identified a small set of proteins that met the FDR threshold (<0.05) for differential abundance, reflecting inter‐patient heterogeneity combined with conservative statistical criteria applied to minimize false positives (Figure S5).

### 3.1 Proteomic Differences Between Primary and Recurrent Samples in Patients

Differential protein analysis between primary and recurrent samples identified a small number of significant proteins in the unsorted and CD45^+^ immune cell fractions. In contrast, no proteins met the significance threshold in the CD45^−^ cell fraction.

In the unsorted samples (Figure S5A), four proteins reached the statistical significance threshold we set for differences in abundance between conditions. ACTBL2 [42], an actin‐related protein associated with cytoskeletal organization; DEFA1 [43], an α‐defensin involved in antimicrobial activity; and MX1 [44], an interferon‐inducible dynamin‐like GTPase, were all significantly downregulated in recurrent samples. In contrast, HMGA2 [45], a chromatin‐associated architectural protein, was significantly upregulated.

In the CD45^+^ immune cell fraction (Figure S5B), only one protein passed the cutoff for the same statistical significance threshold. NUCKS1 [46], a nuclear protein broadly involved in chromatin‐associated processes and cell‐cycle regulation, was significantly downregulated in recurrent samples.

### 3.2 Proteomic and Pathway Alterations Distinguishing Primary and Recurrent Unsorted Cells

**FIGURE 4:**
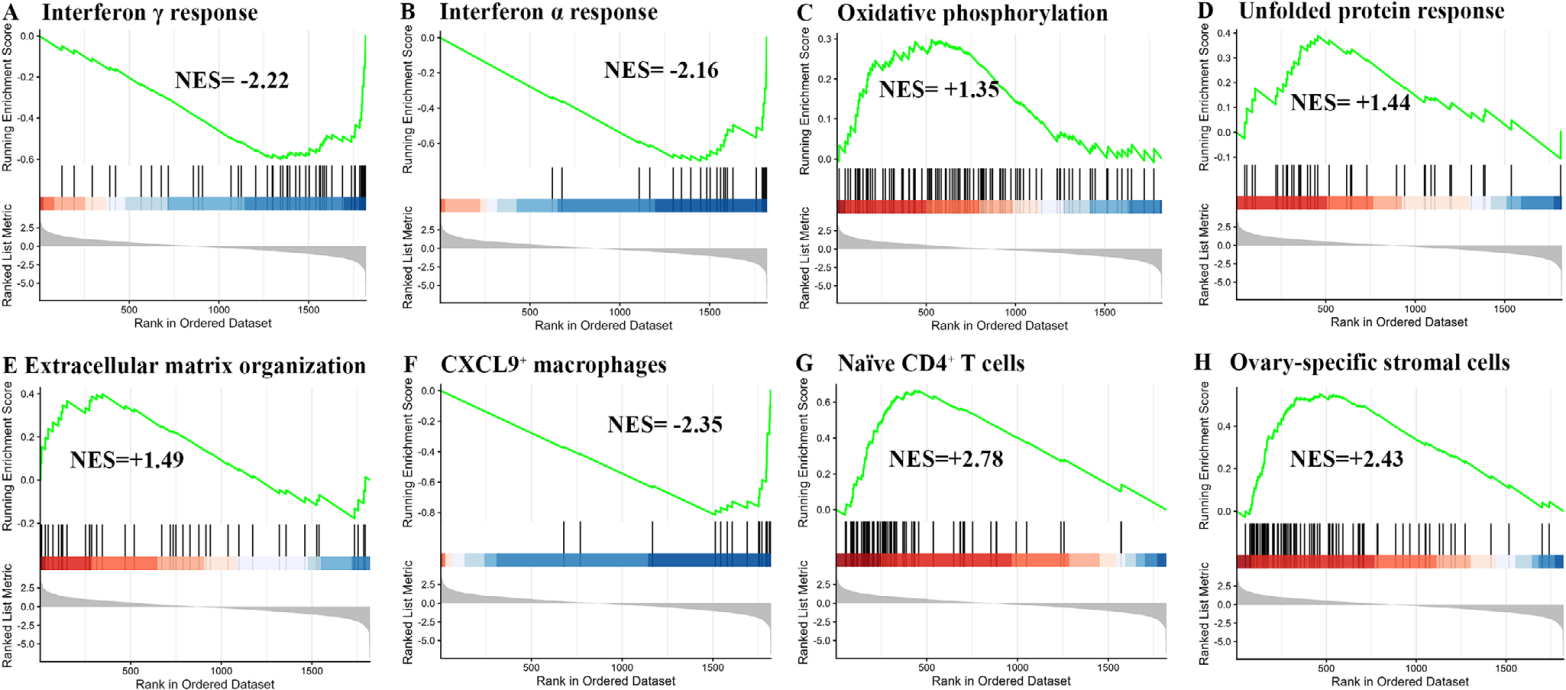
Global immune, metabolic, and stromal remodeling in recurrent unsorted ascites. Gene set enrichment analysis (GSEA) was performed on ranked differences in protein expression between paired recurrent and primary unsorted ascites samples, where the green line indicates running enrichment score, and vertical black bars represent positions of leading‐edge genes in the ranked list with NES being the normalized enrichment score. Immune suppression in recurrent samples is characterized by significant downregulation of (A) the Hallmark interferon γ response and (B) the interferon α response pathways. Metabolic and stress adaptation is supported by positive enrichment of (C) the Hallmark oxidative phosphorylation (OXPHOS) and (D) unfolded protein response (UPR) signatures. Microenvironmental remodeling is highlighted by enrichment of (E) the Reactome extracellular matrix (ECM) organization pathway, suggesting increased fibrosis. Compartment‐specific alterations inferred using C8 cell type signature gene sets reveal depletion of (F) CXCL9^+^ macrophages concurrent with enrichment of (G) naïve CD4^+^ T cells and (H) ovary‐specific stromal cells in recurrent samples.

Gene Set Enrichment Analysis (GSEA) of the ranked protein expression differences in the unsorted cell fraction revealed significant alterations in the global ascites fluid cellular microenvironment, characterized by distinct immune and metabolic shifts. Using the MSigDB Hallmark gene sets, we observed a profound suppression of immune signaling in recurrent samples. The Interferon γ Response (NES = –2.22) and Interferon α Response (NES = –2.16) were the most downregulated pathways (Figure 4A,B), with leading‐edge subsets including key antiviral mediators such as MX1, STAT1, STAT2, and IFIT2. Patient‐paired protein abundances for these representative interferon‐stimulated proteins were compared between primary and recurrent samples (Figure S6A–D). The Oxidative Phosphorylation (OXPHOS) signature was enriched (NES = +1.35) (Figure 4C). This shift was defined by the upregulation of mitochondrial components, including TOMM70, SLC25A5, and ATP5PO. Representative leading‐edge proteins contributing to this pathway are shown in patient‐paired comparisons (Figure S6E–G). Additionally, the Unfolded Protein Response (UPR) was positively enriched (NES = +1.44) (Figure 4D), involving stress‐response proteins such as SRPRB and NPM1 (Figure S6H,I). Beyond cellular pathways, analysis using the Reactome database highlighted structural changes in the ascites environment, specifically an enrichment of the Extracellular Matrix (ECM) Organization pathway (NES = +1.49) in recurrent samples (Figure 4E). Key drivers of this signature included remodeling proteins such as FN1 (Fibronectin), COL1A1 (Collagen Type I), and Cathepsins (CTSB, CTSD) (Figure S6J–M). Finally, enrichment analysis using cell‐type‐specific gene sets (C8 database) revealed compartment‐specific compositional changes. Recurrent samples showed a marked depletion of the CXCL9+ Macrophage signature (NES = –2.35) (Figure 4F). Inspection of the leading‐edge subset confirmed that the downregulation of key proteins such as STAT1 and GBP1 (Figure S6B,N) caused this suppression. Conversely, signatures for Naive CD4^+^ T cells (NES = +2.78) (Figure 4G) and Ovary‐specific Stromal Cells (NES = +2.43) were significantly enriched (Figure 4H), with the latter characterized by high expression of ATP1A1 and ribosomal proteins (Figure S6O).

### 3.3 Proteomic and Pathway Alterations Distinguishing Primary and Recurrent CD45^−^ Cells

**FIGURE 5:**
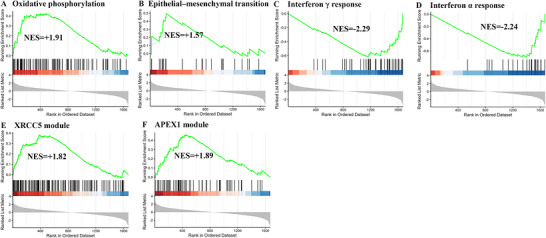
Intrinsic metabolic, phenotypic, and molecular reprogramming in the recurrent CD45^−^ fraction. GSEA was performed on the CD45^−^ fraction to identify alterations, where the green line indicates running enrichment score, and vertical black bars represent positions of leading‐edge genes in the ranked list with NES being the normalized enrichment score. Including (A) upregulation of the Hallmark oxidative phosphorylation (OXPHOS) pathway in recurrent samples, (B) enrichment of the epithelial–mesenchymal transition signature, and suppression of (C) the Hallmark interferon γ response and (D) the interferon α response pathways. Analysis of computational gene sets (C4 database) further revealed upregulation of DNA repair and stress response modules, specifically (E) the XRCC5 module and (F) the APEX1 module.

We next examined the non‐immune CD45^−^ fraction to identify intrinsic alterations. GSEA using the Hallmark gene sets revealed striking metabolic and phenotypic reprogramming in the recurrent tumor‐enriched fraction. The most significantly enriched pathway was oxidative phosphorylation (OXPHOS) (NES = +1.91) (Figure 5A), driven by the upregulation of mitochondrial ATP synthase subunits (ATP5PF, ATP5PB) and fatty acid oxidation enzymes such as HADHB. These representative mitochondrial proteins were further evaluated at the individual protein level (Figure S7A–C). Concurrently, the Epithelial‐Mesenchymal Transition signature was positively enriched (NES = +1.57) (Figure 5B), characterized by elevated levels of mesenchymal markers and collagen‐modifying enzymes, including VIM (Vimentin), CALU (Calumenin), and PLOD2/3 (Figure S7D–G). In contrast, immune recognition pathways were potently suppressed. Similar to the global microenvironment, the Interferon γ Response (NES = –2.29) and Interferon α Response (NES = –2.24) were the most downregulated pathways (Figure 5C,D), characterized by the depletion of interferon‐stimulated genes, including ISG15, WARS1, and MT2A. Representative interferon‐stimulated proteins associated with this depletion were further evaluated at the individual protein level (Figure S7H–J). To identify robust signatures, we analyzed the C4 database, which comprises computational gene sets derived from extensive collections of cancer‐related expression data. This analysis identified the enrichment of specific gene modules associated with DNA repair and stress response. Specifically, the XRCC5 module (NES = +1.82) and APEX1 module (NES = +1.89) were significantly upregulated (Figure 5E,F). The leading edge of these signatures included key components of the non‐homologous end joining complex, such as XRCC5, XRCC6, and PRKDC, as well as the stress‐response protein HNRNPAB (Figure S7K–N).

### 3.4 Proteomic and Pathway Alterations Distinguishing Primary and Recurrent CD45^+^ Cells

**FIGURE 6:**
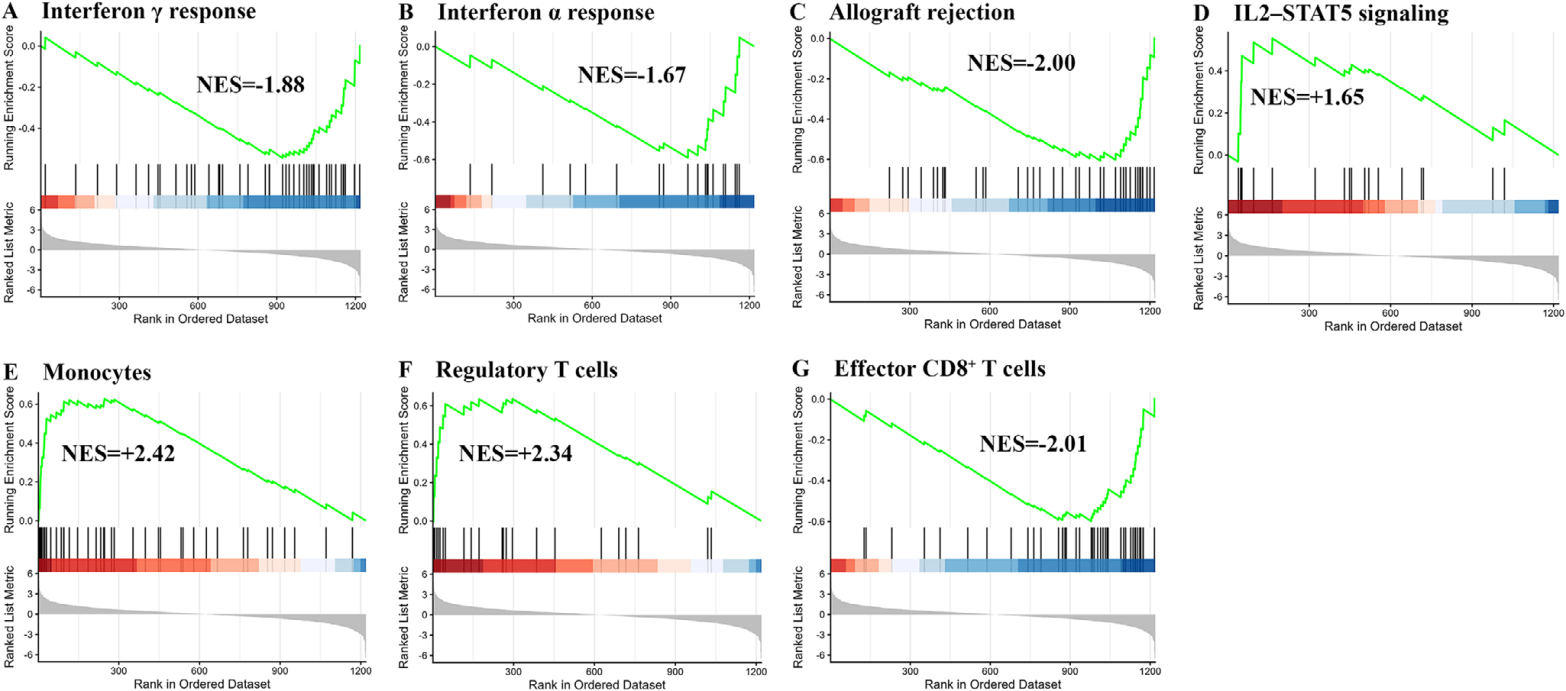
Compartment‐specific pathway alterations in the recurrent CD45^+^ immune‐enriched fraction. GSEA was performed on ranked protein expression differences in the CD45^+^ fraction, where the green line indicates running enrichment score, and vertical black bars represent positions of leading‐edge genes in the ranked list with NES being the normalized enrichment score. Immune suppression in recurrent samples is characterized by downregulation of (A) the Hallmark interferon γ response and (B) the interferon α response pathways, along with suppression of (C) the allograft rejection signature. Additional immune modulation is supported by positive enrichment of (D) the Hallmark IL2–STAT5 signaling pathway, (E) C7 monocyte signatures, and (F) Treg signatures, accompanied by suppression of (G) the effector CD8^+^ T cell signature.

Finally, we analyzed the CD45^+^ immune‐enriched fraction to characterize compartment‐specific immunological alterations. GSEA using Hallmark gene sets revealed a profound suppression of inflammatory signaling within the immune compartment. Mirroring the global downregulation observed in the unsorted and CD45^−^ fractions, the Interferon γ Response (NES = –1.88) and Interferon α Response (NES = –1.67) pathways were significantly suppressed (Figure 6A,B). The Allograft Rejection pathway was the most strongly downregulated signature (NES = –2.00) (Figure 6C), characterized by the depletion of antigen processing and presentation machinery, including TAP1 and TAP2. Representative antigen‐processing proteins were further evaluated at the individual protein level (Figure S8A–B). While inflammatory responses were dampened, the IL2–STAT5 signaling pathway was positively enriched (NES = +1.65), driven by the upregulation of downstream targets such as CD44 and CTSZ (Figure 6D). Representative proteins associated with this pathway were further evaluated at the individual protein level (Figure S8C–D). To further dissect immune cell states, we applied the C7 immunologic signature database. This analysis uncovered a distinct phenotypic shift in the myeloid compartment. Contrasting with the depletion of pro‐inflammatory, M1‐like CXCL9^+^ macrophages observed in the unsorted fraction, the CD45^+^ fraction displayed a strong compensatory enrichment of monocyte‐related signatures (e.g., GSE29618_MONOCYTE_VS_MDC_UP, NES = +2.42) (Figure 6E). Together, these features are consistent with a shift toward a potential immunosuppressive macrophage/monocyte state. The leading edge of this signature was driven by high expression of CD14 and the canonical M2‐like macrophage marker CD163 (Figure S8E,F). Concurrently, Treg signatures were significantly enriched (NES = +2.34) (Figure 6F), in parallel with the increased IL2–STAT5 signaling activity. In contrast, signatures associated with Effector CD8^+^ T cell function were suppressed (e.g., GSE41978_ID2_KO_VS_BIM_KO_KLRG1_LOW_EFFECTOR_CD8_TCELL_UP, NES = –2.01) (Figure 6G), marked by the downregulation of key effector support proteins such as STAT1, TAPBP, and PTPRC (Figure S8G–I).

## 4 Discussion

In this study, ascites‐derived cells from patients with HGSOC were analyzed to characterize proteomic changes associated with disease recurrence. The recurrent samples were collected after prior platinum‐based chemotherapy; however, because the treatment regimens were not identical across the three patients (Table S1) and the cohort is small, this treatment history is provided only as clinical context and not as a basis for treatment‐specific conclusions. Given that inter‐patient variability can obscure consistent signals in clinical samples, conventional unpaired t‐tests may be insufficient to capture coherent biological trends in heterogeneous clinical datasets [47]. To address this heterogeneity [1], we employed GSEA [40], which identifies coordinated pathway‐level changes rather than relying on individual protein differences. This approach enables a more robust interpretation of recurrence‐related molecular alterations in heterogeneous patient populations.

Although the number of proteins showing statistically significant changes in relative expression between primary and recurrent samples was limited, the changes observed in the immune cell–enriched fractions point to biological differences that may accompany disease recurrence. Within the CD45^+^ immune fraction, we observed a reduced abundance of NUCKS1 [46], which is a chromatin‐associated protein that is involved in DNA repair and cell cycle progression. In the context of immunity, NUCKS1 is essential for sustaining the rapid proliferation required for lymphocyte activation and clonal expansion [48, 49]. Consequently, the downregulation of NUCKS1 suggests a compromised proliferative capacity in recurrent immune cells, further supporting the possibility of immune functional attenuation. By contrast, the absence of significant differences in protein relative expression in the CD45^−^ fraction, which comprises a heterogeneous mixture of non‐hematopoietic cell types that are tumor‐enriched, may indicate that large‐scale proteomic changes are less evident within the tumor‐enriched compartment itself and perhaps occur more subtly or over a longer term in that compartment. This is compatible with evidence that immune components of the ascites environment play a substantial role in ovarian cancer progression and relapse [50, 51].

Expanding on these findings, our GSEA analysis suggests that the molecular landscape of the unsorted fraction likely represents a convergent integration of the CD45^−^ and CD45^+^ compartments. Importantly, rather than presenting conflicting trends, the GSEA data across these three fractions appear to be mutually reinforcing. For instance, the pervasive suppression of interferon responses observed in the unsorted fraction was mirrored in both sorted fractions, supporting the notion that this represents a recurrent and convergent feature of the tumor microenvironment at disease recurrence, despite inter‐patient heterogeneity. Although the overall trends were largely concordant across patients, one patient exhibited a more attenuated or partially divergent response in certain pathways rather than a complete reversal, showing the inherent inter‐patient heterogeneity of the tumor microenvironment (Figures S6A,C,D, and S7E, F). Nevertheless, the cell‐type‐resolved analysis provided insight into critical compartment‐specific details that might otherwise be undetectable in analyses of proteomes from bulk cell mixtures. By dissecting these fractions, we could more directly associate specific adaptations, such as intrinsic metabolic reprogramming and DNA repair mechanisms, with the tumor compartment, while inferring distinct phenotypic polarization states within the immune population.

**FIGURE 7:**
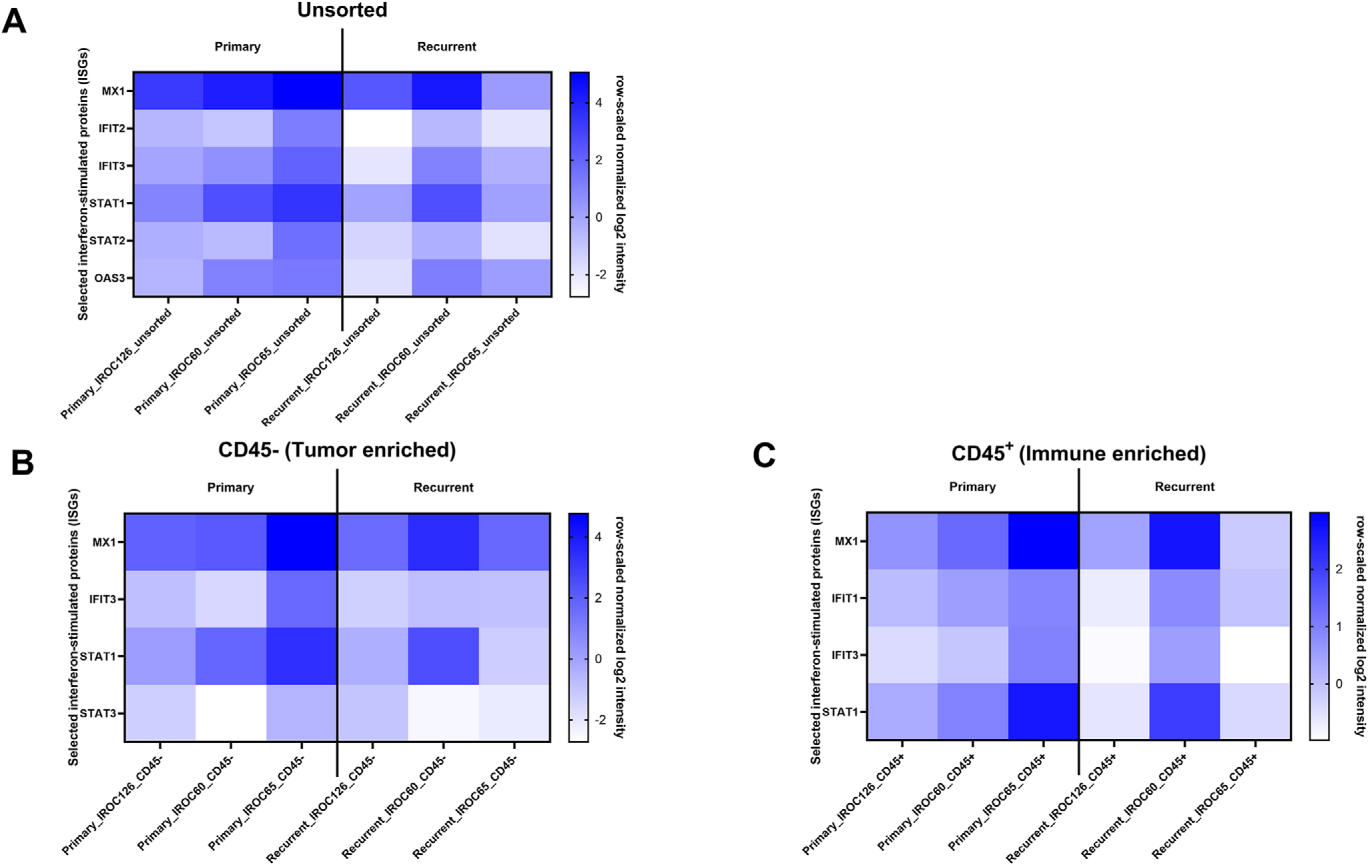
Cell‐type–resolved expression patterns of interferon‐stimulated proteins (ISGs) in primary (collected before chemotherapy) and recurrent ascites samples (collected upon disease recurrence). Heatmaps display selected interferon‐stimulated proteins (ISGs) across (A) unsorted ascites cells, (B) CD45^−^ tumor‐enriched fraction, and (C) CD45^+^ immune‐enriched fraction. Samples are grouped by disease stage (primary vs. recurrent) across three patients. Across all three compartments, a general decrease in ISG‐associated signal intensity is observed in recurrent samples relative to primary samples. While individual proteins and patients show variability, this trend is consistently reflected across multiple ISGs, including signal transducer and activator of transcription (STAT) family proteins, interferon‐induced protein with tetratricopeptide repeats (IFIT) family proteins, and myxovirus resistance protein 1 (MX1).

Beyond the general attenuation of interferon signaling across all compartments, closer inspection of the proteins contributing most strongly to the interferon γ response revealed that this reduction was not uniformly distributed. Instead, it repeatedly involved canonical interferon‐stimulated genes (ISGs) [52], including STAT and IFIT family proteins, as well as MX1‐associated machinery. While the exact composition and magnitude of these protein‐level changes varied among the three patients, the recurrent suppression of core ISG components and interferon α/γ pathways points to a shared, pathway‐level convergence rather than a random loss of inflammatory signaling (Figure 7). Interferon‐stimulated gene modules have previously been described in the context of therapy resistance, most notably as the interferon‐related DNA damage resistance signature (IRDS), a gene program associated with resistance to genotoxic stress and radiation [53, 54]. The pattern observed in our cohort is highly reminiscent of these resistance‐associated modules. Subsets of these ISGs have been implicated in promoting resistance to DNA damage [55], showing a potential functional link between interferon signaling dynamics and treatment response. Although our data does not demonstrate the presence of this resistance‐associated signature overall, they cautiously suggest that recurrent ascites exhibits an IRDS‐like tendency. In this setting, overlapping subsets of canonical ISGs are coordinately suppressed across tumor‐enriched, immune‐enriched, and unsorted compartments.

The coordinated downregulation of interferon‐stimulated genes that we observe, exemplified by STAT1 and MX1, is concordant in direction with observations from large public HGSOC resources. Transcriptomic and proteogenomic characterizations of primary HGSOC, including the TCGA and CPTAC cohorts [47, 56], have defined an immunoreactive molecular subtype distinguished by elevated interferon‐ and immune‐related signaling, and higher activity of these programs has generally been associated with more favorable clinical outcomes [57, 58]. Conversely, attenuation of interferon/ISG signaling has been linked to less immunogenic, treatment‐refractory disease [53, 54]. The reduction of STAT1‐ and MX1‐associated interferon signaling that we detect in recurrent ascites is therefore consistent with the direction reported in these population‐level datasets. Although we did not re‐analyze these external cohorts, the agreement between our compartment‐resolved observations and previously published TCGA/CPTAC‐based analyses provides independent, literature‐level support for interferon‐pathway suppression as a recurrence‐associated feature and helps to offset the limited size of our own cohort. Direct orthogonal validation, for example, by immunoblotting or targeted immunoassays for STAT1 and MX1, remains a valuable direction for future work.

GSEA analysis of Hallmark pathways in the unsorted fraction revealed a profound reconfiguration of the ascites microenvironment in recurrent disease. We observed strong negative enrichment of interferon‐α/‐γ response and allograft rejection pathways, suggesting a marked attenuation of anti‐tumor immunity and antigen‐presentation programs. Metabolically, the recurrence‐associated state was characterized not merely by reduced glycolysis, but by a distinct switch toward oxidative phosphorylation (OXPHOS). This metabolic shift may reflect selection accompanying disease recurrence; platinum‐taxane therapy has separately been proposed to eliminate rapidly proliferating glycolytic cells while enriching for slow‐cycling, mitochondrial‐dependent persister states [59, 60]. Enrichment of oxidative phosphorylation (OXPHOS) signatures should be interpreted as a broad oxidative metabolic phenotype rather than activation of a single, well‐defined pathway. While OXPHOS reflects increased mitochondrial electron transport and oxygen consumption, it does not specify which substrates enter the tricarboxylic acid (TCA) cycle or which components of the electron transport chain are preferentially engaged [61]. Such metabolic plasticity has been reported in ovarian cancer and is associated with survival advantages under therapeutic stress [62]. In the context of chemoresistance, this oxidative phenotype is likely linked less to ATP production per se and more to enhanced redox homeostasis. Increased mitochondrial activity has been associated with glutathione‐dependent detoxification and ROS buffering, potentially raising the threshold for chemotherapy‐induced oxidative damage. Consistent with this interpretation, pharmacologic perturbation of mitochondrial metabolism, including inhibition of OXPHOS‐associated processes, has been shown to restore chemosensitivity in preclinical ovarian cancer models partially [63, 64]. Future studies integrating isotope‐tracing approaches, such as 13C–glucose–based metabolic flux analysis coupled with computational modeling frameworks (e.g., INCA), will be required to resolve the specific mitochondrial pathways and substrate flows underlying this oxidative adaptation [65].

Complementing these intracellular shifts, Reactome and cell‐type‐specific (C8 database) analyses identified a significant upregulation of extracellular matrix (ECM) organization and ovary‐specific stromal signatures [66], alongside a depletion of CXCL9^+^ macrophages [67]. Together, these findings are consistent with a recurrence‐associated state characterized by immune evasion, including loss of interferon signaling, metabolic reprogramming toward mitochondrial bioenergetics, and the formation of a fibrotic, cell‐exclusionary physical barrier [68].

Within the tumor‐enriched CD45^−^ fraction, the signatures of elevated OXPHOS and suppressed interferon signaling broadly mirrored those observed in the unsorted fraction analysis. However, inspection of the leading‐edge subsets suggests molecular drivers specific to the tumor compartment. Unlike the broad mitochondrial upregulation seen in the unsorted fraction, the metabolic profile of CD45^−^ cells appeared characterized by the enrichment of catalytic subunits of ATP synthase (ATP5PF, ATP5PB) and enzymes governing fatty acid oxidation (HADHB). This distinction suggests that the recurrent tumor‐enriched fraction may not merely increase mitochondrial mass but may specifically optimize ATP production efficiency and fuel flexibility (e.g., fatty acid utilization) to survive nutrient stress [59, 60]. Unique to this fraction, this metabolic adaptation seems to coincide with active molecular defense mechanisms, specifically the upregulation of DNA repair modules driven by XRCC5 and APEX1. The enrichment of this non‐homologous end joining complex, and base excision repair components could imply an enhanced intrinsic capacity to repair DNA lesions, potentially raising the apoptotic threshold [69]. Furthermore, the concurrent Epithelial‐Mesenchymal Transition signature may indicate that these metabolically resilient cells also possess increased invasive plasticity [22].

While mirroring the global attenuation of interferon signaling noted in the unsorted analysis, the CD45^+^ immune cell compartment revealed a distinct phenotypic reprogramming of the myeloid lineage. The depletion of pro‐inflammatory CXCL9^+^ macrophages observed in the bulk analysis appears to be counterbalanced by an enrichment of immunosuppressive, M2‐like monocyte signatures (driven by CD14, CD163) within the CD45^+^ population [70]. This pattern is consistent with a polarization shift rather than a simple loss of myeloid cells, creating a permissive niche for tumor growth. Furthermore, this suppressive landscape is actively reinforced by the enrichment of Treg signatures and IL‐2–STAT5 signaling [71], alongside a structural defect in the antigen‐presentation machinery (downregulation of TAP1/TAP2) [72]. In addition to these myeloid and regulatory T‐cell alterations, effector CD8^+^ T‐cell signatures were reduced, reflected by lower expression of STAT1, TAPBP, and PTPRC. Taken together, these observations point to a convergent pattern of diminished antitumor immune activity in the recurrent CD45^+^ compartment [73].

A distinguishing feature of the present study is its focus on paired primary and recurrent ascites analyzed at cell‐type resolution. Previous proteomic and proteogenomic studies of HGSOC have provided important insights into the molecular architecture of bulk tumor tissue [47], the identification of chemosensitivity‐associated proteins and candidate therapeutic targets [20], the definition of molecular subgroups, and the proteomic characterization of ascites‐derived tumor cells [21]. These studies have largely examined primary or bulk material, in which tumor‐cell and immune contributions are intermixed. In contrast, our analysis compares matched primary and recurrent ascites from the same patients and resolves each sample into unsorted, CD45^+^ immune‐enriched, and CD45^−^ tumor‐enriched fractions, allowing recurrence‐associated proteomic changes to be assigned to specific cellular compartments. Our observations are broadly consistent with prior reports linking interferon‐related programs to therapy resistance in ovarian cancer, and extend them by showing that the suppression of interferon signaling at recurrence is a convergent feature detectable across tumor‐enriched, immune‐enriched, and unsorted compartments rather than a property of bulk tissue alone. To our knowledge, this compartment‐resolved, paired primary‐versus‐recurrent view of the ascites proteome has not been reported previously and constitutes the principal conceptual novelty of this work.

However, it is imperative to interpret these findings with careful consideration of several significant limitations inherent in this study. A key limitation of this study is the limited cohort size, comprising three patients (*n* = 3). Even though the GSEA results discussed were statistically significant, questions remain about the generalizability of our conclusions due to limited statistical power. The observed trends would need to be validated in a larger, more diverse patient cohort to be considered a relevant mechanism across all patients with recurrent HGSOC.

Second, the BRCA1/2 (Breast Cancer gene 1/2) mutation status for these patients was unavailable. Given the strong linkage between BRCA genotype, DNA repair capacity, and interferon pathway activation, the absence of this information further constrains our ability to distinguish whether the observed IFN‐low signatures reflect intrinsic BRCA‐dependent biology [74].

Methodologically, no dedicated dead‐cell or debris‐depletion step (e.g., FACS‐based live‐cell sorting or a commercial dead‐cell removal kit) was performed prior to MACS; because all paired primary and recurrent samples were processed with an identical thawing, washing, filtration, and enrichment workflow, any residual contribution from non‐viable cells or debris is expected to be systematic rather than specific to a disease state. In addition, fraction purity was not directly measured; although the proteomic profiles of the two fractions were consistent with effective enrichment—with leukocyte markers such as PTPRC, CD14, and CD163 detected in the CD45^+^ fraction and non‐hematopoietic, tumor‐associated proteins such as vimentin in the CD45^−^ fraction—we cannot exclude some degree of cross‐fraction contamination, and orthogonal validation of enrichment (e.g., by flow cytometry) is warranted in future studies.

Overall, recurrent ascites fluid samples from three patients who underwent chemotherapy consistently exhibited suppressed interferon signaling across tumor (CD45^–^), immune (CD45^+^), and unsorted populations, reflecting a broadly immunosuppressive microenvironment [75] and aligning with reports that chemo‐resistant ovarian tumors dampen IFN responses [76]. Tumor‐enriched CD45^−^ cells showed a marked upregulation of oxidative phosphorylation known to accompany chemoresistance [64]. These tumor‐driven metabolic and proliferative programs were also evident in the proteomic data of unsorted cells, indicating that the bulk ascites fluid cellular profile is a composite of both tumor and immune cell trends. Importantly, the three patients examined here had received platinum‐based chemotherapy, although the small cohort and the non‐identical treatment regimens preclude attributing the observed proteomic shifts specifically to treatment. More broadly, chemotherapy is known to deplete fast‐dividing immune effectors, causing lymphopenia and cytokine changes [77], while selecting tumor clones with enhanced mitochondrial and mitotic capacity. Together, these integrated findings suggest that ascites samples from patients with recurrent disease are characterized by tumor‐enriched cells exhibiting increased bioenergetic and metabolic features, within an ascites microenvironment that shows molecular signatures of immune suppression, consistent with a recurrence‐associated adaptive phenotype.

## 5 Conclusion

In summary, this pilot‐scale cell‐type–resolved proteomics analysis of three paired HGSOC ascites cases nominates interferon attenuation and oxidative metabolic reprogramming as recurrence‐associated programs. Among the three chemotherapy‐treated patients, the recurrent tumor‐enriched fraction tended to show enhanced mitochondrial metabolism, notably oxidative phosphorylation. These alterations were accompanied by a general reduction of interferon‐α and interferon‐γ signaling activities across tumor and immune compartments, consistent with immune suppression in recurrent diseases.

## Conflicts of Interest

The authors declare no conflicts of interest.

## Supporting information




**Supporting File 1**: prca70053‐sup‐0001‐SuppMat.docx.

**Supporting File 2**: prca70053‐sup‐0002‐Data.pdf.

**Supporting File 3**: prca70053‐sup‐0003‐TableS1.xls.

## Data Availability

The data that support the findings of this study are openly available in ProteomeXchange Consortium via the PRIDE at https://www.ebi.ac.uk/pride/archive/, reference number PXD073562. The mass spectrometry proteomics data and GSEA results have been deposited to the ProteomeXchange Consortium via the PRIDE [78] partner repository with the dataset identifier (PXD073562).
