# Supplementary material for: Cell‐Type‐Resolved Proteomics Reveals Distinct Immune and Metabolic Programs Between Primary and Recurrent Ascites in HGSOC
Source: Proteomics Clin Appl. 2026 Aug 18;20(5):e70053. doi: 10.1002/prca.70053 (PMC13483348; doi:10.1002/prca.70053)
Supplement: Supplementary file 1 [file PRCA-20-e70053-s001.pdf]

Supporting Information

Supplementary A. Database search parameters used in MSFragger.

**Parameter**	**Value**
**Mass tolerance**	
precursor_mass_lower	−20 ppm
precursor_mass_upper	20 ppm
precursor_true_tolerance	20 ppm
fragment_mass_tolerance	20 ppm
isotope_error	0/1/2/3
**Enzyme and digestion**	
search_enzyme_name_1	stricttrypsin
search_enzyme_cut_1	KR
search_enzyme_sense_1	C
allowed_missed_cleavage_1	2
digest_min_length	7
digest_max_length	50
digest_mass_range	500.0–5000.0 Da
precursor_charge	1–4
max_fragment_charge	2
**Modifications**	
variable_mod_01	15.9949 M (oxidation of methionine), max 3 per peptide
variable_mod_02	42.0106 [^ (protein N-terminal acetylation), max 1 per peptide
add_C_cysteine	57.02146 (carbamidomethylation, fixed)
max_variable_mods_per_peptide	3
max_variable_mods_combinations	5000
**Ion series**	
fragment_ion_series	b, y
**Output**	
output_format	pepXML_pin
output_max_expect	50
