# Supplementary material for: Cell‐Type‐Resolved Proteomics Reveals Distinct Immune and Metabolic Programs Between Primary and Recurrent Ascites in HGSOC
Source: Proteomics Clin Appl. 2026 Aug 18;20(5):e70053. doi: 10.1002/prca.70053 (PMC13483348; doi:10.1002/prca.70053)
Supplement: Supplementary file 2 [file PRCA-20-e70053-s002.docx]

**Figure S1. Robustness of gene set enrichment results to the choice of protein ranking metric.** Normalized enrichment scores (NES) from gene set enrichment analysis (GSEA) were computed using two alternative protein ranking metrics and compared for each ascites fraction: the signed DEqMS moderated t-statistic (x-axis) versus sign(log₂FC) × −log₁₀(p) (y-axis). Each point represents one gene set, and the dashed line denotes the identity (y = x). Panels correspond to the unsorted (left), CD45^−^ (tumor-enriched; middle), and CD45^+^ (immune-enriched; right) fractions. In each panel, ρ is the correlation between the two sets of NES values, and "n/N same direction" denotes the number of gene sets with concordant enrichment signs across both metrics. Representative pathways are highlighted in blue and labeled (e.g., IFN-α, IFN-γ, EMT, OXPHOS, IL2–STAT5). The strong concordance across metrics indicates that the enrichment results are robust to the choice of ranking metric.

**Figure S2. Data-quality control of the unsorted ascites proteome.** (A) Number of proteins quantified per sample (mean ± SD of three technical replicates). (B) Percentage of missing values per sample before imputation (mean ± SD); retained proteins were quantified in ≥12/18 samples. (C) Pearson correlation between samples (KNN-imputed log₂ intensities), ordered by disease stage; the block-diagonal structure reflects higher correlation among technical replicates than across samples. (D) Distribution of log₂ intensities per sample of KNN-imputed values, before and after median normalization. Blue, primary; red, recurrent.

**Figure S3. Data-quality control of the CD45⁻ (tumor-enriched) ascites proteome.** (A) Number of proteins quantified per sample (mean ± SD of three technical replicates). (B) Percentage of missing values per sample before imputation (mean ± SD); retained proteins were quantified in ≥12/18 samples. (C) Pearson correlation between samples (KNN-imputed log₂ intensities), ordered by disease stage; the block-diagonal structure reflects higher correlation among technical replicates than across samples. (D) Distribution of log₂ intensities per sample of KNN-imputed values, before and after median normalization. Blue, primary; red, recurrent.

**Figure S4. Data-quality control of the CD45⁺ (immune-enriched) ascites proteome.** (A) Number of proteins quantified per sample (mean ± SD of three technical replicates). (B) Percentage of missing values per sample before imputation (mean ± SD); retained proteins were quantified in ≥12/18 samples. (C) Pearson correlation between samples (KNN-imputed log₂ intensities), ordered by disease stage; the block-diagonal structure reflects higher correlation among technical replicates than across samples. (D) Distribution of log₂ intensities per sample of KNN-imputed values, before and after median normalization. Blue, primary; red, recurrent.

**Figure S5. Volcano plot of primary versus recurrent unsorted cells (A) and immune cells (B) with proteins reaching significance.**

Unsorted Cells (A): Shows significant proteins: ACTBL2, DEFA1, and MX1 (downregulated, Purple) and HMGA2 (upregulated, Orange). CD45+ Immune Cells (B): Shows NUCKS1 (downregulated, Purple) as the only significant protein. Proteins that do not meet the significance threshold are shown in Grey (NO-DIFF); FDR-adjusted p-value < 0.05 and |log2 foldchange| ≥1).

**Figure S6. Representative protein trends supporting pathway alterations in the unsorted fraction.**

Patient-level expression (log₂ LFQ intensity, mean of three technical replicates) of leading-edge proteins for the pathways enriched in recurrent unsorted ascites, comparing paired primary and recurrent samples for the three patients (IROC126, IROC60, IROC65). Panels are grouped by pathway (labels at left): interferon γ/α response (MX1, STAT1, STAT2, IFIT2; A–D); oxidative phosphorylation (TOMM70, SLC25A5, ATP5PO; E–G); unfolded protein response (SRPRB, NPM1; H, I); extracellular matrix organization (FN1, COL1A1, CTSB, CTSD; J–M); CXCL9⁺ macrophages (GBP1; N; STAT1 in panel B is also a leading-edge protein of this signature); and ovary-specific stromal cells (ATP1A1; O).

**Figure S7. Representative protein trends supporting pathway alterations in the CD45⁻ tumor-enriched fraction.**

Patient-level expression (log₂ LFQ intensity, mean of three technical replicates) of leading-edge proteins for the pathways enriched in recurrent CD45⁻ tumor-enriched fraction, comparing paired primary and recurrent samples for the three patients (IROC126, IROC60, IROC65). Panels are grouped by pathway (labels at left): oxidative phosphorylation (ATP5PF, ATP5PB, HADHB; A–C); epithelial–mesenchymal transition (VIM, CALU, PLOD2, PLOD3; D–G); interferon γ/α response (ISG15, WARS1, MT2A; H–J); and the XRCC5 and APEX1 DNA-repair modules (XRCC5, XRCC6, PRKDC, HNRNPAB; K–N).

**Figure S8. Representative protein trends supporting pathway alterations in the CD45⁺ immune-enriched fraction.**
Patient-level expression (log₂ LFQ intensity, mean of three technical replicates) of leading-edge proteins for the pathways altered in recurrent CD45⁺ immune cells, comparing paired primary and recurrent samples for the three patients (IROC126, IROC60, IROC65). Panels are grouped by pathway (labels at left): allograft rejection / antigen processing (TAP1, TAP2; A, B); IL2–STAT5 signaling (CD44, CTSZ; C, D); monocyte / M2-like macrophage signature (CD14, CD163; E, F); and effector CD8⁺ T cells (STAT1, TAPBP, PTPRC; G–I).
